# Supplementary material for: Neuropeltis acuminata (P. Beauv.): Investigation of the Chemical Variability and In Vitro Anti-inflammatory Activity of the Leaf Essential Oil from the Ivorian Species
Source: Molecules. 2022 Jun 10;27(12):3759. doi: 10.3390/molecules27123759 (PMC9230793; doi:10.3390/molecules27123759)
Supplement: Supplementary file 1 [file molecules-27-03759-s001.zip › molecules-1740921-supplementary.pdf]

Supplementary Materials

Neuropeltis acuminata (P. Beauv.): Investigation of the Chemical Variability and In Vitro Anti-inflammatory Activity of the Leaf Essential Oil from the Ivorian Species

Didjour Albert Kambiré [1,2], Ahmont Claude Landry Kablan [1], Thierry Acafou Yapi [3], Sophie Vincenti [2], Jacques Maury [2], Nicolas Baldovini [4], Pierre Tomi [2], Mathieu Paoli [2], Jean Brice Boti [3] and Félix Tomi [2,*]

[1] UPR de Chimie Organique, Département de Mathématiques, Physique et Chimie, UFR des Sciences Biologiques, Université Péléforo Gon Coulibaly, Korhogo BP 1328, Ivory Coast; dakambire@gmail.com (D.A.K.); kablanahmont@yahoo.fr (A.C.L.K.)

[2] Laboratoire Sciences Pour l'Environnement, Université de Corse—CNRS, UMR 6134 SPE, Route des Sanguinaires, 20000 Ajaccio, France; vincenti_s@univ-corse.fr (S.V.); maury_j@univ-corse.fr (J.M.); tomi_p@univ-corse.fr (P.T.); paoli_m@univ-corse.fr (M.P.)

[3] Laboratoire de Constitution et Réaction de la Matière, UFR-SSMT, Université Félix Houphouët-Boigny, Abidjan BP V34, Ivory Coast; acafouth@yahoo.fr (T.A.Y.); jeanbriceboti@hotmail.fr (J.B.B.)

[4] Institut de Chimie de Nice, CNRS UMR 7272, Université Côte d'Azur, Parc Valrose, CEDEX 2, 06108 Nice, France; nicolas.baldovini@unice.fr

* Correspondence: tomi_f@univ-corse.fr

Figure S1: ^{1}H NMR spectrum of compound **96** in $CDCl_3$ (400 MHz).

Figure S2: ^{13}C NMR spectrum of compound **96** in $CDCl_3$ (100 MHz).

Figure S3: DEPT 135 NMR spectrum of compound **96** in $CDCl_3$ (100 MHz).

Figure S4: DEPT 90 NMR spectrum of compound **96** in $CDCl_3$ (100 MHz).

Figure S5: HSQC spectrum of compound **96** in $CDCl_3$.

Figure S6: COSY spectrum of compound **96** in $CDCl_3$.

Figure S7: HMBC spectrum of compound **96** in $CDCl_3$.

Figure S8: NOESY spectrum of compound **96** in $CDCl_3$.

Table S1: Chemical composition of the 30 leaf essential oil samples from *Neuropeltis acuminata*.

Table S2: Plant material and essential oil extraction data.

Figure S1. ^{1}H NMR spectrum of compound **96** in $CDCl_3$ (400 MHz).

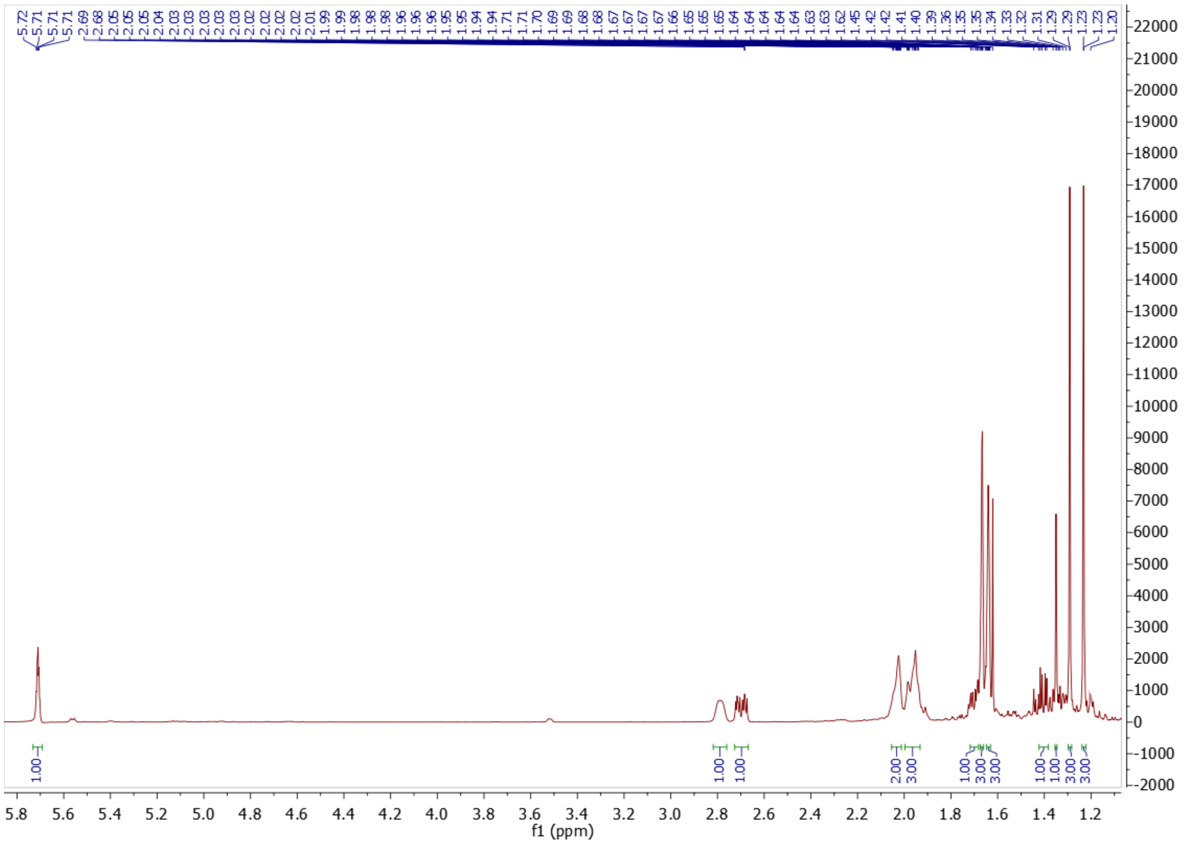

Figure S2.^{13}C NMR spectrum of compound **96** in $CDCl_3$ (100 MHz).

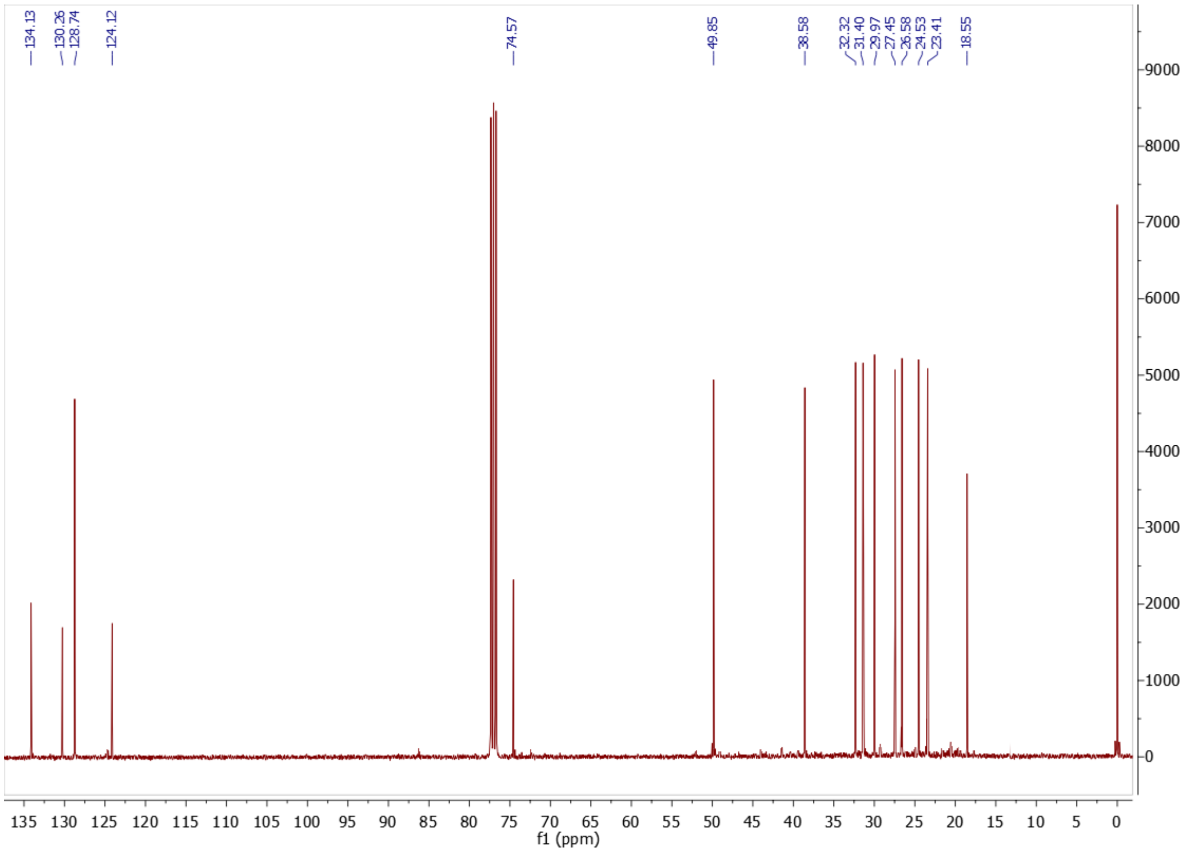

Figure S3. DEPT 135 NMR spectrum of compound **96** in $CDCl_3$ (100 MHz).

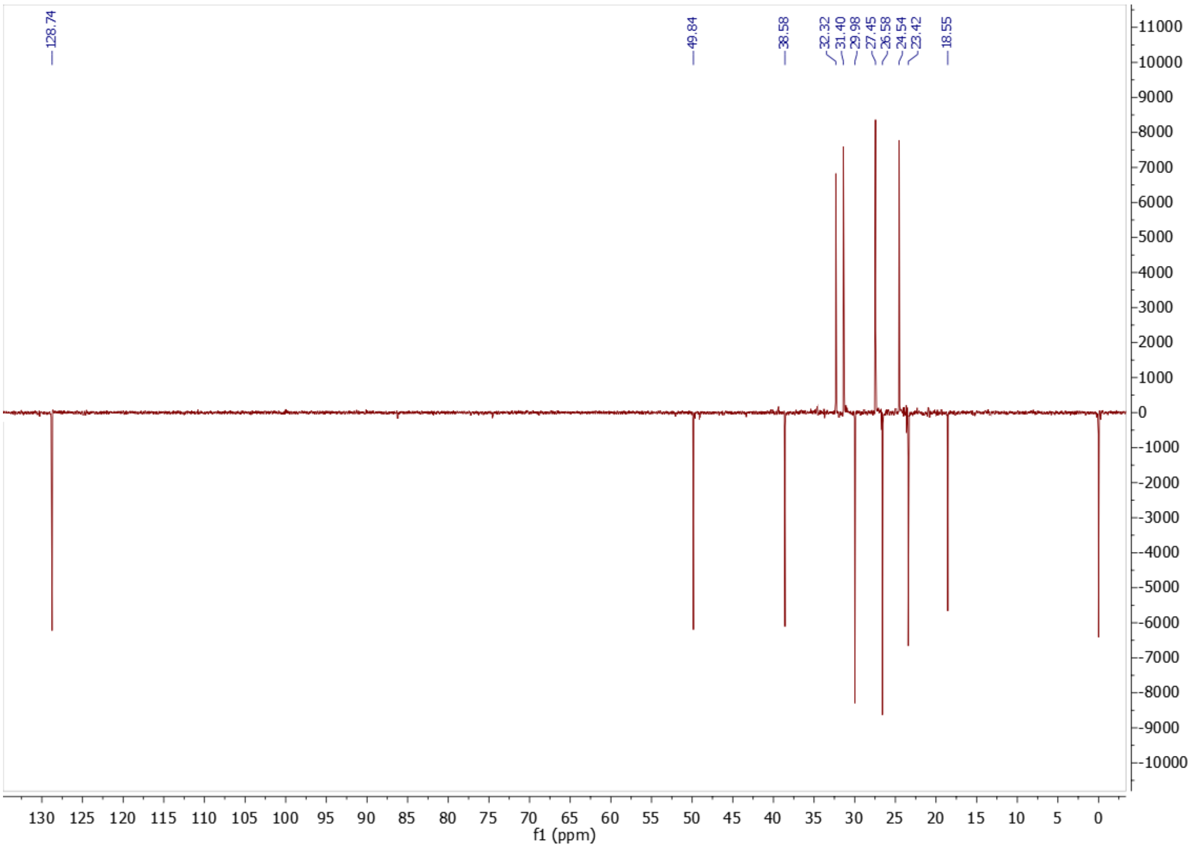

Figure S4. DEPT 90 NMR spectrum of compound **96** in $CDCl_3$ (100 MHz).

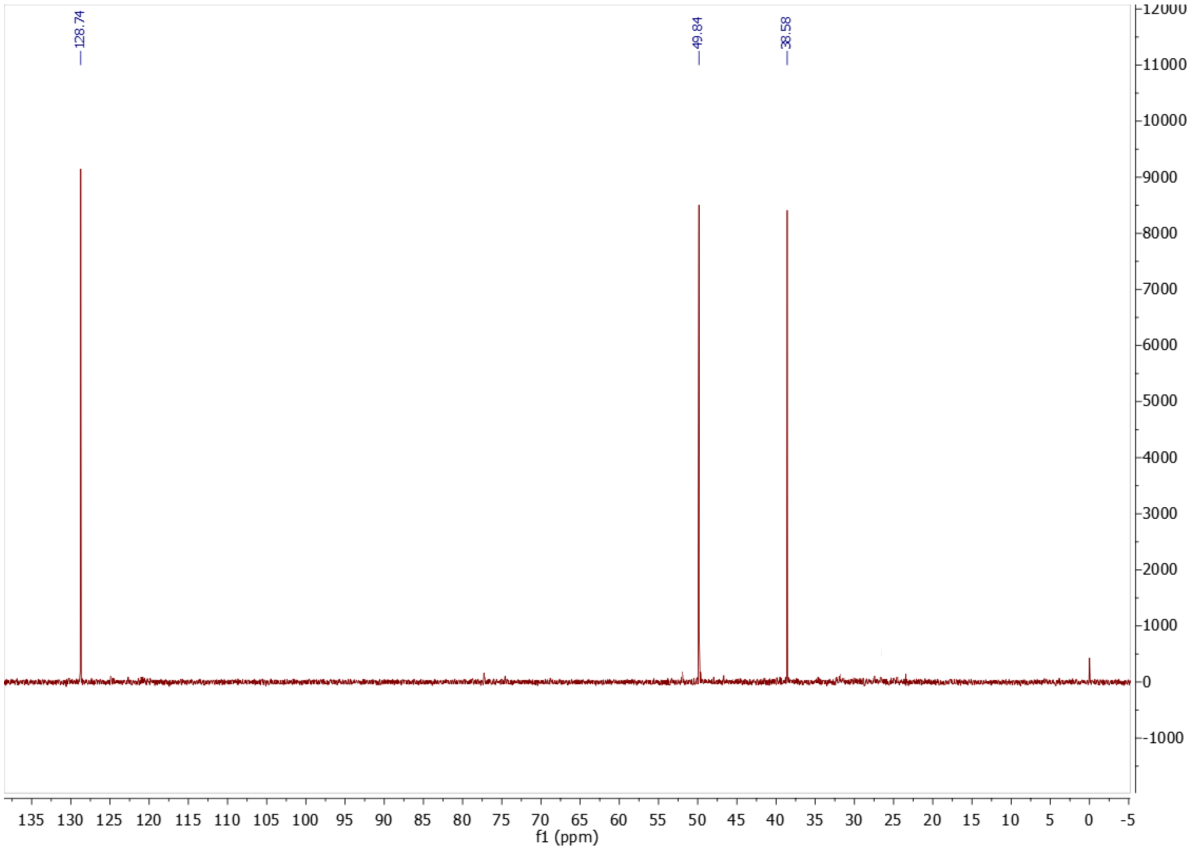

Figure S5. HSQC spectrum of compound **96** in $CDCl_3$.

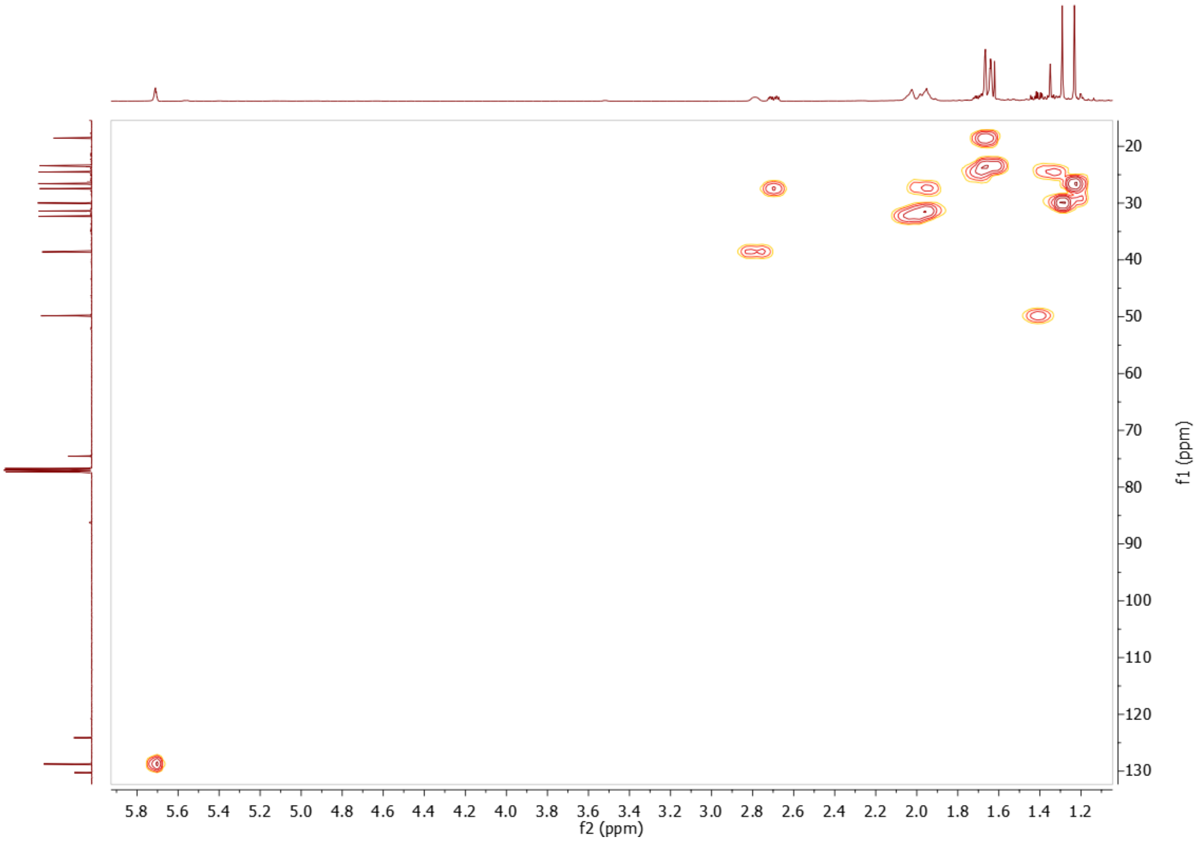

Figure S6. COSY spectrum of compound **96** in $CDCl_3$.

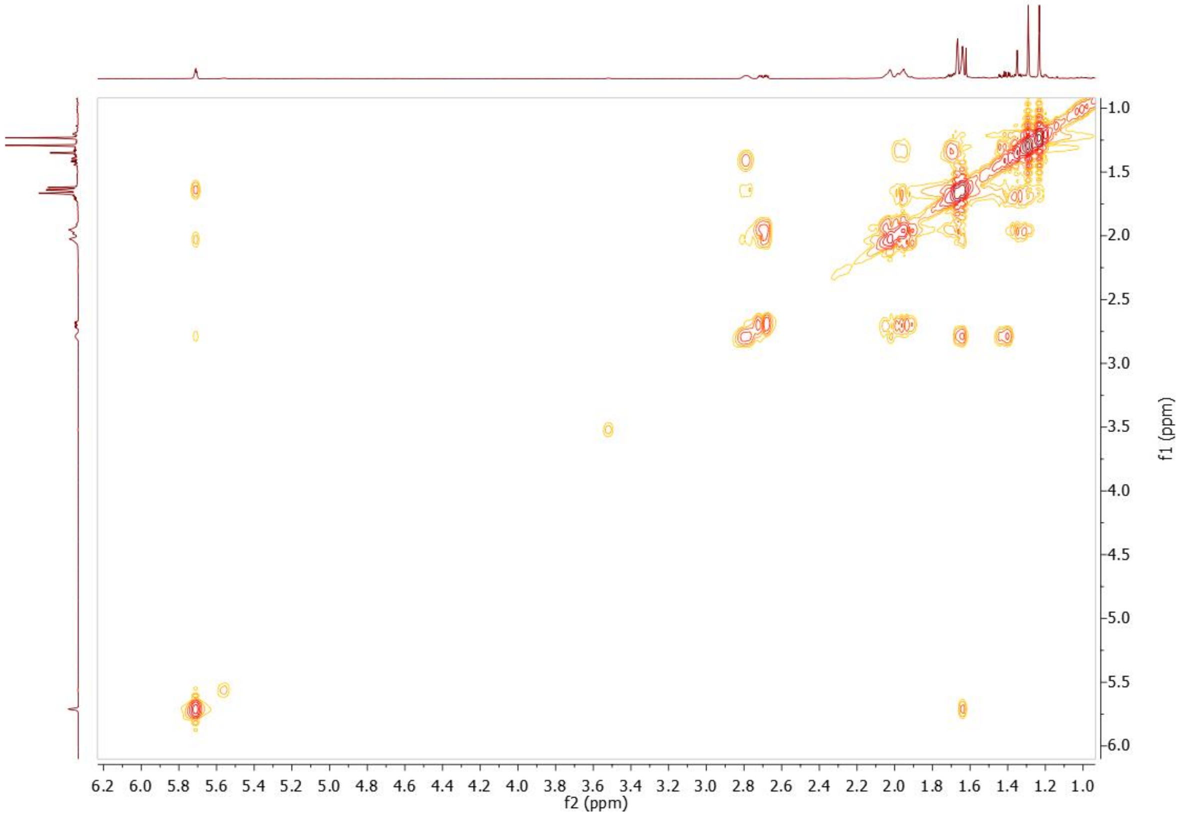

Figure S7. HMBC spectrum of compound **96** in $CDCl_3$.

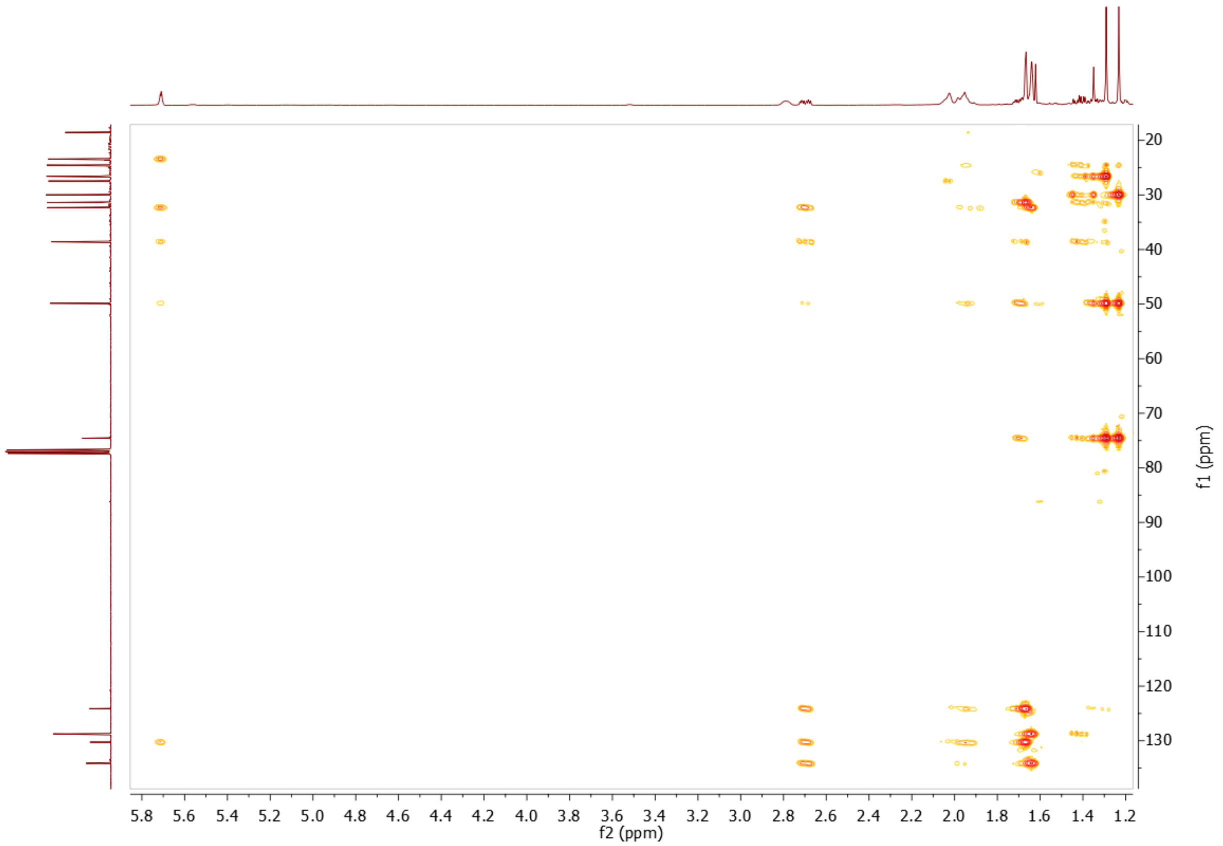

Figure S8. NOESY spectrum of compound **96** in $CDCl_3$.

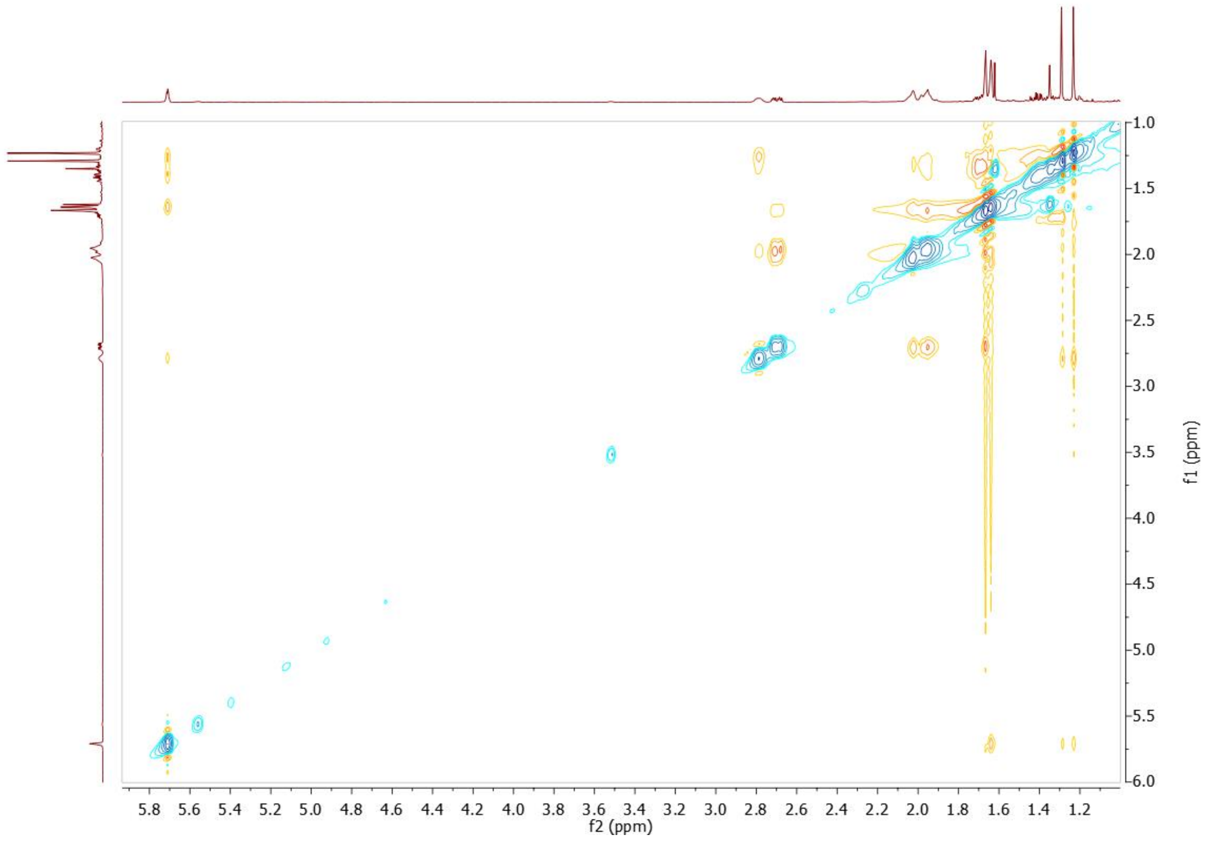

Table S1. Chemical composition of five leaf essential oil samples from *N. acuminata*.

N°	Compounds[a]	RIa	RIp	S1	S2	S3	S30	S4	S5	S6	S7	S8	S10	S11	S12	S13	S14	S15	S17	S19	S9	S16	S18	S20	S21	S22	S23	S24	S25	S26	S27	S28	S29	Identification
1	(*Z*)-Hex-3-en-1-ol	840	1388	-	tr	0.1	0.2	-	-	0.1	0.2	0.2	tr	tr	tr	0.1	0.1	0.1	0.1	tr	-	0.1	0.1	tr	tr	tr	tr	-	tr	tr	tr	tr	tr	RI, MS
2	Hexanol	854	1355	0.1	0.1	0.1	0.1	tr	-	-	0.3	0.3	tr	tr	tr	0.1	0.4	0.2	0.1	tr	0.1	0.2	0.1	0.1	tr	tr	0.1	tr	tr	tr	tr	-	-	RI, MS
3	α-Thujene	923	1020	tr	0.1	0.2	0.2	-	tr	0.2	-	0.1	tr	0.1	-	0.1	0.1	-	0.1	-	tr	tr	-	-	tr	-	tr	0.1	tr	tr	tr	tr	tr	RI, MS, *^{13}C-NMR*
4	α-Pinene	931	1016	0.1	0.3	0.1	1.1	tr	tr	tr	0.1	tr	tr	tr	tr	tr	0.1	0.1	tr	tr	0.1	tr	tr	tr	0.1	0.1	0.1	0.2	0.6	0.2	0.4	0.1	0.3	RI, MS, ^{13}C-NMR
5	Sabinene	966	1127	0.4	1.5	0.6	tr	0.2	0.2	0.3	0.2	0.2	0.6	0.5	0.8	0.5	0.3	0.4	0.4	0.3	0.4	0.1	0.1	0.1	0.4	0.6	0.9	0.6	0.8	1.2	0.9	0.7	0.7	RI, MS, ^{13}C-NMR
6	β-Pinene	971	1116	0.2	1.0	0.4	0.3	tr	tr	tr	0.1	tr	0.1	tr	tr	tr	0.1	0.1	tr	tr	0.4	tr	tr	tr	0.1	0.1	0.2	0.2	0.4	0.3	0.3	0.2	0.2	RI, MS, ^{13}C-NMR
7	Myrcene	981	1166	0.1	0.3	0.1	tr	tr	tr	0.1	0.1	0.1	0.1	tr	tr	-	0.1	0.1	tr	tr	0.1	tr	tr	tr	0.1	0.1	0.1	tr	0.1	0.1	0.1	0.1	0.1	RI, MS
8	α-Terpinene	1010	1186	-	0.2	0.1	0.2	tr	-	-	-	0.1	-	0.1	-	0.1	0.1	-	0.1	-	tr	-	-	-	0.1	-	tr	0.1	tr	tr	tr	-	-	RI, MS
9	β-Phellandrene*	1022	1215	tr	0.1	-	-		-	tr		0.1	tr	-	-	0.1	-	tr	tr	-		tr	-	-	-	0.1	0.1	0.1	0.1	0.1	0.1	tr	0.1	RI, MS
10	Limonene*	1022	1205	0.1	0.3	0.1	0.1	0.1	tr	0.1	0.1	tr	0.1	tr	tr	-	tr	tr	tr	tr	0.1	-	tr	tr	-	0.1	0.1	0.1	0.2	0.2	0.2	0.2	0.1	RI, MS
11	(*Z*)-β-Ocimene	1026	1237	tr	0.1	tr	0.1	0.1	0.1	0.1	0.1	tr	tr	0.1	-	0.1	tr	-	0.1	-	tr	0.1	-	-	tr	-	-	0.3	-	0.1	tr	-	tr	RI, MS, *^{13}C-NMR*
12	(*E*)-β-Ocimene	1037	1255	0.9	2.3	1.2	tr	0.5	0.5	0.8	0.8	0.8	1.1	1.3	0.6	1.5	2.2	1.1	1.2	1.3	0.4	1.8	1.8	1.7	1.1	0.9	1.1	1.2	1.7	1.7	1.4	1.2	1.1	RI, MS, ^{13}C-NMR
13	γ-Terpinene	1049	1250	tr	0.3	tr	0.1	tr	-	0.2	-	tr	tr	0.1	tr	0.1	tr	-	tr	tr	tr	-	-	-	0.1	-	tr	0.2	tr	tr	tr	tr	tr	RI, MS, *^{13}C-NMR*
14	Terpinolene	1079	1288	tr	0.2	0.2	0.1	-	tr	0.1	-	-	-	0.1	-	0.1	0.1	-	0.1	-	tr	-	-	-	0.1	-	tr	0.2	tr	tr	tr	-	-	RI, MS, *^{13}C-NMR*
15	Linalool	1085	1550	0.1	0.2	tr	tr	tr	-	0.1	tr	tr	0.1	0.1	tr	tr	0.1	0.1	tr	tr	tr	tr	tr	0.1	0.1	0.1	0.1	tr	0.1	0.1	0.1	0.1	0.1	RI, MS
16	Terpinen-4-ol	1163	1604	0.1	0.6	0.1	tr	tr	-	0.1	tr	tr	0.1	tr	0.1	tr	-	tr	tr	0.1	0.1	-	-	-	tr	tr	0.1	tr	0.1	0.1	0.1	0.1	0.1	RI, MS, ^{13}C-NMR
17	Neral	1217	1680	0.1	0.1	0.2	0.1	0.1	tr	0.1	-	0.1	-	0.1	tr	0.1	0.1	tr	tr	tr	0.1	-	-	-	-	-	-	0.2	-	-	-	-	-	RI, MS, *^{13}C-NMR*
18	Geraniol	1236	1837	0.1	0.2	0.1	0.1	-		0.2	tr	-	-	tr	-	0.2	0.1	-	0.1	-	-	0.1	-	-	tr	-	tr	0.1	tr	-	-	-	-	RI, MS, *^{13}C-NMR*
19	Geranial	1244	1732	0.1	0.2	0.1	0.1	tr	0.1	0.1	-	0.1	-	0.1	tr	0.1	0.1	0.1	tr	tr	0.1	tr	-	-	-	-	-	0.1	-	-	-	-	-	RI, MS
20	Thymol	1268	2190	1.1	2.2	1.3	0.1	tr	tr	tr	tr	tr	0.1	tr	tr	tr	tr	0.1	0.1	0.1	tr	tr	0.1	tr	tr	tr	tr	tr	0.1	tr	0.1	tr	0.1	RI, MS, ^{13}C-NMR
21	Carvacrol	1277	2228	-	tr	0.1	0.2	0.1	0.2	0.1	0.1	0.2	0.1	0.1	0.1	tr	tr	0.1	0.1	0.1	0.1	tr	tr	-	0.1	tr	tr	-	-	tr	-	tr	-	RI, MS
22	Cogeijerene	1282	1540	tr	tr	0.1	0.1	-	tr	0.1	-	tr	-	0.1	-	0.2	0.1	-	tr	-	-	-	-	tr	0.1	0.1	0.1	tr	0.1	0.1	0.1	tr	0.1	RI, MS, *^{13}C-NMR*
23	Bicycloelemene	1332	1483	tr	-	0.1	tr	-	0.1	tr	-	0.1	tr	0.1	0.1	0.1	0.2	0.1	0.1	0.1	0.1	tr	tr	-	0.3	0.4	0.2	0.1	0.2	0.2	0.2	0.2	0.2	RI, MS
24	δ-Elemene	1335	1472	0.4	0.3	0.4	0.2	0.3	0.1	0.2	0.4	0.3	0.8	2.0	0.6	2.2	3.0	1.4	1.5	1.8	tr	0.3	0.9	0.2	3.1	2.6	2.6	2.6	2.7	2.7	2.8	2.7	2.5	RI, MS, ^{13}C-NMR
25	α-Cubebene	1348	1459	-	tr	0.1	0.1	tr	tr	0.2	0.1	tr	0.2	0.1	0.1	tr	0.2	0.2	0.1	0.2	0.5	-	0.1	-	tr	tr	tr	0.1	0.1	tr	0.1	tr	tr	RI, MS, ^{13}C-NMR
26	Cyclosativene	1369	1483	0.3	0.3	0.1	tr	tr	-	0.1	-	-	tr	tr	-	0.1	tr	tr	0.1	0.1	tr	tr	-	-	0.6	0.4	0.1	0.1	0.3	tr	0.1	tr	0.2	RI, MS, ^{13}C-NMR
27	α-Ylangene	1371	1468	tr	-	0.1	tr	tr	-	0.1	tr	-	-	tr	-	0.1	0.2	-	tr	-	-	-	-	tr	0.1	0.1	0.1	-	0.1	tr	0.1	0.1	0.1	RI, MS
28	α-Copaene	1375	1493	0.3	0.4	0.1	0.7	0.1	0.1	0.1	0.2	0.1	0.2	0.5	0.1	0.1	0.3	0.7	0.1	0.2	0.1	tr	0.1	tr	0.3	0.2	0.1	tr	0.2	0.1	0.1	0.1	0.1	RI, MS, ^{13}C-NMR
29	β-Bourbonene	1383	1520	tr	tr	0.1	0.2	tr	tr	0.1	-	-	0.1	0.1	0.1	tr	0.1	0.1	tr	0.1	-	-	tr	-	tr	tr	tr	0.1	tr	tr	tr	tr	tr	RI, MS
30	β-Cubebene*	1387	1539	0.2	0.2	0.2	0.3	0.2	0.2	0.3	0.5	0.3	0.7	0.3	0.3	0.4	0.8	0.9	0.7	0.7	0.1	0.1	0.1	0.1	1.0	1.0	1.0	1.0	1.1	1.0	1.0	1.0	0.9	RI, MS, ^{13}C-NMR
31	β-Elemene*	1387	1591	4.5	4.1	4.4	5.2	1.1	0.5	0.6	1.0	0.6	1.8	1.5	1.1	1.6	1.9	3.7	3.0	3.0	2.8	2.7	2.1	4.6	1.7	1.5	1.2	1.2	1.4	1.2	1.5	1.1	1.5	RI, MS, ^{13}C-NMR
32	Cyperene	1399	1528	0.2	0.4	0.2	0.3	tr	-	0.1	tr	tr	tr	tr	-	0.2	tr	tr	tr	tr	-	-	-	-	0.1	0.1	0.1	tr	0.1	tr	0.1	tr	0.1	RI, MS, *^{13}C-NMR*
33	α-Gurjunene	1409	1531	0.7	0.6	0.7	2.3	0.6	0.1	tr	0.2	0.1	0.4	0.1	0.1	0.2	0.5	2.8	2.7	2.4	0.1	0.5	0.9	0.1	tr	tr	tr	tr	tr	tr	tr	tr	tr	RI, MS, ^{13}C-NMR
34	(*E*)-β-Caryophyllene	1417	1597	1.6	2.4	1.8	4.7	0.9	0.9	3.4	9.4	2.0	15.9	21.9	25.0	20.0	12.5	14.4	15.8	15.5	45.4	34.2	32.5	34.4	1.1	1.1	1.0	1.0	1.1	1.1	1.2	1.0	1.1	RI, MS, ^{13}C-NMR
35	β-Copaene	1426	1591	0.9	0.1	0.5	0.1	0.3	0.4	0.1	0.4	0.3	0.5	0.6	0.2	0.7	1.3	0.5	0.6	0.5	0.2	0.1	0.2	0.1	2.9	2.5	3.3	3.1	3.5	3.4	3.6	3.4	3.0	RI, MS, ^{13}C-NMR
36	γ-Elemene #	1427	1640	5.8	4.5	5.5	4.1	0.7	0.2	0.3	0.5	0.3	3.2	1.4	1.5	1.3	0.9	1.2	1.2	1.4	0.6	0.3	0.9	0.3	2.0	1.8	1.1	1.1	1.2	0.9	1.0	0.8	1.3	RI, MS, ^{13}C-NMR
37	*trans*-α-Bergamotene	1432	1586	0.1	0.2	0.1	1.7	0.1	0.1	tr	0.1	0.1	tr	tr	tr	tr	0.2	0.1	tr	tr	tr	tr	tr	tr	0.2	0.1	0.1	0.4	0.2	0.1	0.1	0.1	0.1	RI, MS, ^{13}C-NMR

38	a-Guaiene	1435	1591	0.1	0.1	tr	tr	0.5	0.4	0.1	0.2	0.4	0.1	0.1	tr	-	0.2	0.1	0.3	0.2	0.1	0.1	0.1	tr	0.3	0.3	0.4	0.2	0.4	0.4	0.4	0.4	0.4	RI, MS, ^{13}C-NMR
39	Sesquisabinene A	1436	1647	tr	tr	tr	7.6	tr	tr	tr	tr	tr	tr	tr	tr	tr	tr	tr	tr	tr	tr	tr	tr	tr	0.3	0.3	0.4	0.6	0.4	0.4	0.4	0.4	0.4	RI, MS, ^{13}C-NMR
40	Guaia-6,9-diene	1437	1606	0.1	0.1	tr	0.6	0.1	-	tr	0.1	0.1	tr	0.3	tr	0.1	0.1	0.3	0.6	0.5	0.1	0.1	0.2	0.1	0.7	0.7	0.6	0.3	0.5	0.3	0.3	0.3	0.3	RI, MS, ^{13}C-NMR
41	b-Gurjunene (Calarene)	1444	1591	0.1	0.2	0.1	0.2	tr	-	0.1	tr	tr	tr	0.3	0.1	0.1	0.2	0.3	0.2	0.2	tr	tr	tr	0.1	0.8	0.7	0.6	0.2	0.5	0.4	0.3	0.4	0.3	RI, MS, ^{13}C-NMR
42	(*E*)-β-Farnesene	1446	1661	0.1	0.1	0.1	3.0	0.3	0.4	0.4	0.3	0.4	0.1	0.1	0.1	0.1	0.2	0.1	0.1	0.1	0.1	tr	0.1	tr	0.5	0.4	0.4	0.6	0.3	0.4	0.4	0.4	0.4	RI, MS, ^{13}C-NMR
43	α-Humulene	1450	1670	6.0	5.0	5.8	9.7	0.6	0.5	1.6	1.8	0.6	1.4	2.9	3.0	2.7	2.0	1.1	5.2	1.7	6.3	31.2	10.9	27.1	1.4	1.2	1.3	1.7	0.5	1.3	1.5	1.3	1.3	RI, MS, ^{13}C-NMR
44	cis-β-Bergamotene	1452	1671	0.3	0.3	0.3	3.8	0.2	0.2	0.2	0.2	0.1	0.2	0.2	0.1	0.2	tr	tr	0.1	0.2	0.8	0.4	0.3	1.1	0.1	0.1	0.1	tr	0.1	0.1	tr	0.1	tr	RI, MS, ^{13}C-NMR
45	*allo*-Aromadendrene	1456	1640	0.2	tr	0.2	tr	tr	0.2	tr	0.2	0.5	0.5	0.1	0.2	0.1	0.3	1.3	0.9	1.4	0.1	0.3	0.8	0.2	0.1	tr	0.1	0.1	tr	tr	0.2	0.1	0.1	RI, MS, ^{13}C-NMR
46	Ishwarane	1461	1645	0.2	0.2	0.1	tr	-	-	tr	tr	-	-	0.1	-	0.3	0.1	tr	0.1	-	-	-	-	-	0.2	-	tr	-	-	tr	-	tr	-	RI, MS, *^{13}C-NMR*
47	γ-Muurolene	1469	1688	0.2	0.3	0.1	0.7	0.1	0.1	tr	0.1	0.2	0.4	0.4	0.3	0.2	0.3	0.5	0.3	0.4	0.2	0.1	0.2	0.1	0.2	0.2	0.2	0.1	0.2	0.2	0.2	0.2	0.1	RI, MS, ^{13}C-NMR
48	4,5-*diepi*-Aristolochene	1471	1705	-	tr	0.3	0.1	-	-	-	-	tr	0.2	0.3	0.1	tr	0.2	0.1	tr	0.1	tr	-	tr	-	tr	tr	-	-	-	tr	tr	-	-	RI, MS, *^{13}C-NMR*
49	1,11-Oxidocalamenene	1472	1883	1.0	0.9	1.0	0.4	0.8	0.6	0.4	0.4	0.5	0.7	0.5	0.3	0.5	0.6	tr	1.5	1.2	2.2	1.4	0.8	2.7	0.1	0.1	0.1	0.1	0.1	0.1	0.1	0.1	tr	RI, MS, ^{13}C-NMR
50	Germacrene D	1475	1709	3.1	2.4	3.0	5.6	1.3	0.5	1.1	2.0	1.1	3.4	5.0	3.0	5.2	5.8	5.2	3.2	3.6	2.4	1.0	2.3	1.0	14.4	10.7	9.0	10.2	11.8	9.7	10.2	9.6	9.7	RI, MS, ^{13}C-NMR
51	*trans*-β-Bergamotene	1478	1684	0.1	0.1	0.1	0.7	0.1	0.2	tr	-	0.2	tr	-	-	tr	-	-	tr	-	tr	0.2	tr	-	0.2	0.2	0.2	0.1	0.2	0.2	0.2	0.2	0.1	RI, MS, ^{13}C-NMR
52	β-Selinene	1481	1718	0.5	0.6	0.5	1.3	0.6	0.7	0.3	0.5	0.5	0.3	0.2	0.1	0.2	0.4	0.3	0.3	0.4	0.3	0.2	0.4	0.4	0.3	0.4	0.3	0.3	0.4	0.4	0.4	0.3	0.3	RI, MS, ^{13}C-NMR
53	Furanodiene #	1482	1873	0.1	1.6	0.4	0.4	0.6	0.1	0.1	0.3	0.5	tr	0.4	tr	0.4	0.2	0.3	0.2	0.2	0.6	0.4	0.2	0.3	2.9	5.9	5.4	3.9	2.2	3.6	4.3	3.3	4.9	RI, MS, ^{13}C-NMR
54	Furano-elemene (Curzerene) #	1484	1873	tr	0.2	0.1	tr	tr	-	tr	-	tr	0.1	0.3	tr	0.1	0.8	0.2	0.6	0.2	0.2	0.2	tr	tr	0.1	tr	tr	0.1	0.1	0.1	0.1	tr	-	RI, MS, ^{13}C-NMR
55	4-*epi*-Cubebol	1487	1871	0.1	0.2	0.2	tr	0.6	0.5	0.4	0.7	0.6	0.4	0.2	tr	-	0.1	0.3	tr	-	-	-	tr	-	0.1	0.1	tr	-	0.1	0.1	0.1	0.1	0.1	RI, MS, ^{13}C-NMR
56	Bicyclogermacrene	1490	1732	0.9	0.9	0.9	0.2	0.8	0.6	0.7	0.7	0.8	1.4	0.6	0.5	0.7	0.9	3.2	2.0	3.9	0.2	0.5	1.9	0.3	0.3	0.1	0.1	0.1	0.1	0.1	0.1	0.1	0.1	RI, MS, ^{13}C-NMR
57	α-Selinene	1491	1723	0.7	0.6	0.7	1.7	0.4	0.4	0.2	0.5	0.4	0.7	0.4	0.4	0.4	0.2	0.8	0.3	0.7	0.3	0.2	0.4	0.2	0.5	1.6	2.7	2.2	1.5	2.7	2.8	2.7	2.3	RI, MS, ^{13}C-NMR
58	α-Muurolene	1494	1705	2.9	2.2	2.8	0.1	3.0	3.4	2.2	2.5	2.3	2.5	1.1	2.1	1.0	0.7	0.2	1.1	0.6	10.9	4.6	2.8	6.6	0.1	tr	tr	0.3	tr	0.1	0.1	0.1	tr	RI, MS, ^{13}C-NMR
59	β-Bisabolene	1500	1727	0.8	0.6	0.8	1.2	1.1	1.1	0.9	1.0	1.0	0.6	0.4	0.8	0.4	0.4	0.5	1.9	1.6	0.3	0.5	0.5	0.2	1.7	1.5	1.5	1.5	1.8	1.5	1.6	1.6	1.5	RI, MS, ^{13}C-NMR
60	Cubebol	1505	1885	tr	0.1	tr	0.4	0.1	0.1	tr	0.2	0.1	1.3	0.7	0.6	0.7	0.5	1.8	0.1	0.3	tr	tr	0.4	tr	0.3	tr	tr	0.2	0.2	tr	tr	tr	0.2	RI, MS, ^{13}C-NMR
61	γ-Cadinene	1507	1758	0.2	0.1	0.1	tr	0.1	tr	-	0.1	0.1	-	-	-	0.1	tr	-	0.1	-	-	-	tr	-	0.2	0.2	0.2	0.1	0.2	0.2	0.2	0.2	-	RI, MS
62	(*Z*)-γ-Bisabolene	1510	1732	0.2	-	0.1	-	0.1	0.1	tr	tr	0.1	tr	tr	tr	tr	0.1	0.1	0.1	0.2	0.1	0.1	tr	0.1	0.5	0.6	-	0.1	0.3	-	-	-	0.4	RI, MS, ^{13}C-NMR
63	δ-Cadinene	1514	1758	2.3	2.8	2.4	1.3	0.6	0.5	0.8	1.2	0.7	4.2	8.0	1.8	8.4	12.0	7.4	4.7	2.9	0.4	1.0	1.4	0.9	7.3	7.0	7.5	7.0	7.8	7.4	7.5	7.6	6.5	RI, MS, ^{13}C-NMR
64	Kessane	1521	1761	5.5	4.2	5.2	4.4	0.1	0.3	2.3	2.7	2.5	9.4	14.0	32.5	11.5	1.4	6.2	2.9	4.6	1.0	0.5	0.4	2.7	-	-	-	-	-	-	-	-	-	RI, MS, ^{13}C-NMR
65	(*E*)-γ-Bisabolene	1522	1758	1.1	1.0	1.1	0.2	1.2	1.7	0.4	0.9	0.4	1.0	tr	0.8	tr	0.1	0.1	tr	0.1	tr	tr	0.5	tr	4.1	3.0	3.2	3.4	4.1	3.3	3.3	3.5	3.0	RI, MS, ^{13}C-NMR
66	Selina-4(15),7(11)-diene	1528	1778	0.5	0.5	0.5	0.3	11.2	11.1	8.3	9.3	7.4	4.3	tr	0.8	0.1	0.3	0.1	0.5	2.0	2.2	0.1	1.8	0.1	0.3	0.2	0.2	0.2	0.2	0.2	tr	0.1	0.2	RI, MS, ^{13}C-NMR
67	β-Elemol	1534	2079	0.6	0.8	0.6	tr	5.0	4.9	4.4	4.2	3.9	2.9	1.2	1.9	1.2	1.0	2.0	3.1	4.0	1.7	0.9	2.9	0.9	2.5	2.1	1.6	1.8	2.0	1.6	1.5	1.6	1.6	RI, MS, ^{13}C-NMR
68	Selina-3,7(11)-diene	1537	1778	0.6	0.5	0.2	tr	0.3	0.2	tr	0.2	0.3	0.2	0.3	0.1	tr	tr	0.1	-	0.2	0.1	-	0.1	tr	tr	0.5	0.6	0.2	0.1	0.4	0.3	0.3	0.3	RI, MS, ^{13}C-NMR
69	*cis*-Cadinene ether	1545	1860	0.1	tr	-	-	tr	-	-	0.1	0.1	tr	tr	0.1	0.4	-	-	-	-	-	-	-	-	-	0.1	0.2	tr	-	0.1	0.1	tr	tr	RI, MS, *^{13}C-NMR*
70	(*E*)-Nerolidol	1548	2042	0.4	0.7	0.5	0.6	25.7	30.8	23.3	15.8	23.3	6.6	1.5	0.8	1.6	1.8	0.5	tr	2.6	3.2	0.3	7.8	0.7	0.2	0.2	0.2	0.2	0.2	0.2	0.2	0.2	0.2	RI, MS, ^{13}C-NMR
71	Germacrene B #	1551	1829	16.6	11.3	15.5	10.2	tr	tr	0.9	0.9	tr	4.8	3.5	4.1	3.2	2.2	3.0	3.0	3.5	1.5	0.7	2.2	0.7	5.1	4.8	2.9	3.5	3.2	2.3	3.2	2.3	3.7	RI, MS, ^{13}C-NMR
72	Palustrol	1561	1924	1.6	1.0	1.5	0.2	0.4	tr	1.2	2.8	1.0	0.3	0.1	0.1	0.2	0.5	2.2	2.0	1.7	0.1	0.4	0.6	0.1	tr	tr	tr	tr	tr	tr	tr	tr	tr	RI, MS, ^{13}C-NMR
73	*cis*-Sesquisabinene hydrate	1564	2081	0.1	0.2	0.1	0.4	0.1	0.1	tr	0.1	0.1	1.4	0.8	1.7	0.6	tr	2.8	0.2	0.5	tr	tr	0.3	0.1	0.1	tr	tr	tr	tr	tr	tr	tr	tr	RI, MS, ^{13}C-NMR
74	Caryophyllene oxide	1570	1978	0.1	0.3	0.1	0.4	tr	tr	tr	0.2	tr	0.1	0.3	0.2	0.3	0.3	0.1	0.2	0.1	0.4	0.7	0.3	2.5	tr	tr	tr	tr	tr	tr	tr	tr	tr	RI, MS, ^{13}C-NMR
75	Curzerenone	1575	2025	-	1.6	-	0.1	0.2	0.2	-	0.1	0.7	0.2	0.2	0.1	-	0.3	0.3	0.1	0.2	0.2	0.1	0.2	0.1	tr	tr	-	0.3	-	tr	-	-	-	RI, MS, ^{13}C-NMR
76	7-*epi*-*cis*-Sesquisabinene hydrate	1576	2099	tr	1.5	0.3	0.2	tr	tr	tr	tr	tr	tr	0.6	tr	0.7	1.3	0.1	tr	0.1	tr	0.1	0.1	tr	0.1	tr	0.1	0.1	0.1	0.1	0.1	0.1	0.1	RI, MS, ^{13}C-NMR
77	Viridiflorol	1581	2081	1.0	0.7	0.9	0.1	0.3	tr	tr	0.1	tr	tr	tr	0.1	0.1	0.3	1.4	1.3	1.1	tr	0.4	0.5	0.2	0.3	0.3	0.2	0.2	0.2	0.2	0.2	0.2	0.2	RI, MS, ^{13}C-NMR
78	Guaiol	1584	2088	0.7	0.3	0.6	tr	13.8	13.8	12.2	11.5	10.9	6.5	2.0	2.0	2.3	3.3	2.7	7.8	7.1	2.9	1.9	3.5	1.8	6.9	9.8	12.1	11.6	10.8	13.0	13.0	13.2	12.4	RI, MS, ^{13}C-NMR
79	Ledol*	1593	2025	13.2	9.5	12.5	7.3	1.9	tr	0.3	0.6	tr	0.7	0.5	0.4	1.3	4.5	6.7	8.2	4.5	0.7	2.5	2.9	1.0	0.6	0.3	0.1	0.2	0.3	tr	0.1	tr	0.2	RI, MS, ^{13}C-NMR

80	Copaborneol*	1593	2183	1.2	0.2	1.4	0.1	0.1	-	tr	0.1	tr	-	-	tr	0.1	0.2	0.1	0.1	tr	0.1	tr	tr	-	-	-	tr	0.2	0.1	tr	-	tr	-	RI, MS, ^{13}C-NMR
81	Eudesm-5-en-11-ol	1595	2132	tr	tr	tr	0.1	0.2	0.2	0.2	0.3	0.1	0.7	0.3	0.4	0.3	0.5	tr	0.3	0.2	0.4	0.5	0.2	tr	1.7	1.2	0.5	0.8	1.1	0.4	0.6	0.5	0.8	RI, MS, ^{13}C-NMR
82	*neo*-Intermedeol	1599	2146	0.4	0.1	0.1	-	0.1	-	tr	tr	-	-	0.4	0.1	tr	0.7	0.2	0.1	-	-	0.1	-	tr	tr	-	-	0.2	-	-	-	-	-	RI, MS, ^{13}C-NMR
83	*epi*-Cubenol	1606	2048	tr	0.1	tr	tr	0.4	tr	0.2	tr	0.1	0.2	0.2	0.2	0.2	0.3	0.7	5.5	3.3	0.5	1.0	0.4	0.4	0.5	0.4	0.2	0.3	0.3	tr	0.2	0.2	0.3	RI, MS, ^{13}C-NMR
84	Alismol	1610	2248	0.3	0.2	tr	tr	0.1	tr	-	0.1	0.2	0.1	0.2	0.1	tr	0.6	0.1	tr	0.2	0.2	0.2	0.1	0.5	0.1	0.2	tr	0.1	0.3	0.2	0.2	0.2	0.2	RI, MS, ^{13}C-NMR
85	Eremoligenol	1614	2196	0.6	0.6	0.1	0.2	0.1	tr	-	0.1	0.1	0.2	0.1	tr	-	0.2	0.2	0.1	-	tr	0.1	0.1	tr	0.2	0.1	tr	0.2	0.2	0.1	0.2	0.1	0.2	RI, MS, ^{13}C-NMR
86	10-*epi*-γ-Eudesmol	1617	2096	1.5	0.9	1.4	tr	0.7	0.6	0.3	0.6	0.6	0.9	0.9	0.9	1.3	3.0	1.2	1.3	1.0	0.2	0.4	0.3	tr	0.4	0.4	0.4	0.3	0.5	0.4	0.4	0.4	0.5	RI, MS, ^{13}C-NMR
87	τ-Cadinol	1625	2175	0.7	0.9	0.3	0.3	0.2	0.1	-	0.2	-	1.0	0.3	0.2	0.2	0.3	0.2	0.3	0.3	0.1	0.2	0.2	tr	0.6	0.2	0.2	0.4	0.5	0.5	0.4	0.4	0.4	RI, MS, ^{13}C-NMR
88	τ-Muurolol	1628	2184	0.7	0.9	0.7	tr	0.6	0.4	0.2	0.4	tr	1.7	0.8	0.3	0.8	0.9	2.0	0.6	0.8	0.1	0.2	0.5	tr	0.6	0.7	0.6	0.7	0.7	0.7	0.7	0.7	0.7	RI, MS, ^{13}C-NMR
89	α-Muurolol	1630	2212	0.2	0.2	0.2	tr	tr	tr	tr	0.3	0.4	1.0	0.7	0.4	0.6	0.4	1.0	0.4	0.5	0.2	tr	0.1	0.1	0.2	0.2	0.2	0.2	0.3	0.2	0.2	0.2	0.2	RI, MS, ^{13}C-NMR
90	β-Eudesmol	1634	2225	0.2	0.2	0.2	tr	1.9	2.2	6.3	2.7	2.4	1.5	2.1	1.1	1.9	1.3	1.3	0.9	1.6	0.9	0.5	1.2	0.7	0.6	0.6	0.5	0.5	0.6	0.5	0.5	0.5	0.6	RI, MS, ^{13}C-NMR
91	α-Cadinol	1637	2228	1.1	1.5	1.2	0.4	tr	tr	0.7	tr	tr	tr	tr	tr	0.5	2.4	2.5	1.2	0.5	tr	0.5	tr	tr	tr	tr	tr	tr	tr	tr	tr	tr	tr	RI, MS, ^{13}C-NMR
92	α-Eudesmol	1638	2216	tr	0.1	tr	tr	tr	tr	2.1	tr	tr	1.9	0.7	0.4	0.6	tr	tr	tr	0.2	tr	tr	tr	tr	tr	tr	tr	tr	tr	tr	tr	tr	tr	RI, MS, ^{13}C-NMR
93	Atractylone	1639	2121	0.4	3.2	1.0	tr	3.2	2.8	3.3	2.6	3.2	0.9	2.9	tr	2.7	2.0	3.3	0.8	2.6	1.5	0.8	2.1	1.5	4.8	9.3	11.9	10.0	7.1	10.4	10.1	10.7	12.1	RI, MS, ^{13}C-NMR
94	Intermedeol	1641	2249	0.1	0.3	0.1	tr	1.1	1.2	0.8	0.8	1.0	0.6	0.2	0.1	0.2	0.3	0.3	0.8	0.7	tr	0.2	0.4	0.2	0.7	tr	tr	tr	tr	tr	tr	tr	tr	RI, MS, ^{13}C-NMR
95	Bulnesol*	1651	2207	0.4	0.5	0.4	0.2	7.5	7.6	5.2	5.9	6.1	3.7	1.4	1.6	1.5	1.9	1.7	4.2	3.9	1.6	1.0	3.0	1.0	3.2	5.1	6.6	6.2	5.4	7.0	6.2	7.2	6.4	RI, MS, ^{13}C-NMR
96	δ-Cadinen-11-ol*	1651	2271	0.7	1.4	0.8	0.1	0.2	tr	2.9	3.4	2.1	tr	0.2	tr	0.2	0.4	0.4	0.1	0.2	0.1	tr	tr	0.1	3.1	2.7	1.8	1.8	2.1	1.4	1.5	1.4	2.1	RI, MS, ^{13}C-NMR
97	α-Bisabolol	1666	2208	0.3	0.2	0.3	0.2	tr	0.1	tr	0.2	0.8	tr	0.6	1.6	0.5	0.1	tr	tr	tr	tr	tr	tr	0.1	0.2	0.1	0.1	0.6	0.2	0.1	0.1	0.2	0.1	RI, MS, ^{13}C-NMR
98	*epi*-α-Bisabolol	1668	2214	0.3	0.2	0.2	tr	0.1	0.1	-	0.1	0.5	0.2	-	tr	0.2	-	-	0.2	0.3	tr	-	tr	-	0.6	0.4	0.5	0.1	0.5	0.6	0.3	0.5	0.5	RI, MS, ^{13}C-NMR
99	Cadina-1(10),4-dien-8α-ol	1671	2306	-	-	-	tr	tr	tr	0.4	tr	tr	tr	7.1	0.1	8.1	12.1	1.2	0.2	0.1	tr	1.6	0.5	0.4	0.6	0.4	0.2	0.3	0.2	0.2	0.2	0.1	0.3	RI, MS, ^{13}C-NMR
100	Germacrone	1673	2221	0.7	1.2	0.8	-	0.2	0.2	0.2	0.3	0.3	0.3	tr	0.1	tr	0.1	0.3	0.2	1.2	0.2	tr	0.6	0.1	0.1	0.3	0.2	0.1	0.1	0.2	0.2	0.2	0.3	RI, MS, ^{13}C-NMR
101	(*E*)-γ-Bisabolen-12-al	1761	2348	0.6	1.1	0.7	tr	1.3	1.6	1.1	0.9	3.3	0.4	tr	0.2	tr	0.1	0.1	tr	0.1	0.2	tr	0.3	tr	1.2	1.5	2.0	1.9	2.0	2.4	1.9	2.5	2.0	RI, MS, ^{13}C-NMR
102	(*E*)-γ-Bisabolen-12-ol	1776	2549	6.1	5.9	6.1	5.2	3.6	4.6	2.5	2.7	8.8	1.0	1.1	1.6	1.0	0.8	1.3	0.4	0.5	1.0	0.3	1.4	0.3	9.4	7.4	6.6	7.1	8.4	7.5	5.3	8.1	5.8	RI, MS, ^{13}C-NMR
103	(*E*)-Phytol	2098	2609	0.1	0.1	0.4	5.9	0.2	0.1	0.3	0.2	0.2	0.2	0.2	0.2	0.1	0.2	0.3	0.2	0.2	0.3	0.2	0.3	0.1	0.6	0.2	0.3	0.1	0.6	0.3	0.4	0.4	0.5	RI, MS, ^{13}C-NMR
	Monoterpene hydrocarbons			1.8	6.7	3.0	2.2	0.9	0.8	1.9	1.5	1.4	2.0	2.3	1.4	2.6	3.1	1.8	2.0	1.6	1.5	2.0	1.9	1.8	2.1	2.0	2.6	3.3	3.9	3.9	3.4	2.5	2.6	
	Oxygenated monoterpenes			1.6	3.5	1.9	0.6	0.2	0.3	0.7	0.1	0.4	0.4	0.4	0.2	0.4	0.4	0.4	0.3	0.3	0.4	0.1	0.1	0.1	0.2	0.1	0.2	0.4	0.3	0.2	0.3	0.2	0.3	
	Sesquiterpene hydrocarbons			58.3	47.6	55.4	72.9	27.0	25.3	26.2	38.2	24.3	59.5	67.0	80.1	62.2	50.3	57.1	53.8	55.1	77.3	80.0	65.1	81.7	53.1	46.6	43.5	43.8	46.9	42.8	45.6	42.7	42.6	
	Oxygenated sesquiterpenes			35.5	39.5	34.8	17.3	71.1	72.8	68.7	58.8	71.7	37.5	29.5	17.7	31.3	44.1	39.5	43.0	42.0	19.5	16.9	31.9	15.8	43.0	50.1	52.5	50.7	46.9	51.9	49.0	52.9	53.4	
	Other compounds			0.2	0.2	0.6	6.2	0.2	0.1	0.4	0.7	0.7	0.2	0.2	0.2	0.3	0.7	0.6	0.4	0.2	0.4	0.5	0.5	0.2	0.6	0.2	0.4	0.1	0.6	0.3	0.4	0.4	0.5	
	**Total**			97.4	97.5	95.7	99.2	99.4	99.3	97.9	99.3	98.5	99.6	99.4	99.6	96.8	98.6	99.4	99.5	99.2	99.1	99.5	99.5	99.6	99.0	99.0	99.2	98.3	98.6	99.1	98.7	98.7	99.4	

[a]Order of elution and percentages are given on an apolar column (BP-1), except components with an asterisk (*), where percentages are taken on a polar column (BP-20). (#) Thermolabile compound, percentage evaluated by a combination of GC-FID and ^{13}C-NMR data. RIa, RIp: retention indices measured on apolar and polar capillary column, respectively. (-): not detected; tr: traces level (<0.05%). ^{13}C-NMR: compounds identified by NMR in the essential oil samples and obvious in at least one fraction of chromatography; *^{13}C-NMR (italic)*: compounds identified by NMR in fractions of chromatography.

Table S2. Plant material and essential oil extraction data.

Samples	Leaves weight (g)	Essential oil weight (mg)	Extraction yield (%)	Harvest site	Month	Season
1	101.3	1042.5	1.03	Station 1	January 2021	Dry
2	90.6	921.1	1.02	Station 1	January 2021	Dry
3	270.9	2016.2	0.74	Station 1	January 2021	Dry
4	250.1	2214.8	0.89	Station 2	February 2021	Dry
5	271.4	1678.7	0.62	Station 2	February 2021	Dry
6	239.9	2034.9	0.85	Station 2	February 2021	Dry
7	250.0	2333.3	0.93	Station 2	February 2021	Dry
8	271.6	1659.8	0.61	Station 2	February 2021	Dry
9	268.3	1816.0	0.68	Station 4	February 2021	Dry
10	227.2	1886.6	0.83	Station 3	March 2021	Dry
11	257.0	1499.1	0.58	Station 3	March 2021	Dry
12	214.1	1910.5	0.89	Station 3	March 2021	Dry
13	187.2	1406.1	0.75	Station 3	March 2021	Dry
14	196.7	1925.7	0.98	Station 3	March 2021	Dry
15	202.3	1333.7	0.66	Station 3	March 2021	Dry
16	197.2	1370.0	0.70	Station 4	February 2021	Dry
17	199.9	1148.2	0.57	Station 3	March 2021	Dry
18	178.8	1203.9	0.67	Station 4	February 2021	Dry
19	226.5	1654.1	0.73	Station 3	March 2021	Dry
20	219.0	1875.1	0.86	Station 4	February 2021	Dry
21	228.0	1550.2	0.68	Station 5	March 2021	Dry
22	214.5	1634.5	0.76	Station 5	March 2021	Dry
23	224.2	1324.6	0.59	Station 5	March 2021	Dry
24	206.9	1790.5	0.87	Station 5	March 2021	Dry
25	214.8	1575.6	0.73	Station 5	March 2021	Dry
26	248.00	2344.1	0.95	Station 6	March 2021	Dry
27	259.60	2019.8	0.78	Station 6	March 2021	Dry
28	221.20	1891.7	0.86	Station 6	March 2021	Dry
29	195.10	1933.4	0.99	Station 6	March 2021	Dry
30	212.90	1913.1	0.90	Station 1	January 2021	Dry

Harvest sites locations: Bossématié forest, Region of Abengourou, Eastern Ivory Coast, Station 1 (6°29′26.0″ N and 3°29′11.7″ W). Haut-Sassandra forest, Western Ivory Coast, Station 2 (6°53′40.2″ N and 6°55′36.3″ W), Station 3 (6°57′08.5″ N and 6°59′00.5″ W), Station 4 (6°54′52.7″ N and 6°57′21.1″ W). Yapo-Abbé forest, Southern Ivory Coast, Station 5 (5°41′08.0″ N and 4°06′31.7″ W) and Station 6 (5°41′48.7″ N and 4°05′31.0″ W).
